# Physiological response and survival of Atlantic mackerel exposed to simulated purse seine crowding and release

**DOI:** 10.1093/conphys/coab076

**Published:** 2021-09-14

**Authors:** Neil Anders, Bjørn Roth, Mike Breen

**Affiliations:** 1 Fish Capture Division, Institute of Marine Research (IMR), Bergen, 5817, Norway; 2 Department of Processing Technology, NOFIMA, Stavanger, 4068, Norway

## Abstract

Understanding how animals physiologically respond to capture and release from wild capture fishing is fundamental for developing practices that enhance their welfare and survival. As part of purse seine fishing for small pelagic fish in northern European waters, excess and/or unwanted catches are routinely released from the net in a process called slipping. Due to excessive crowding in the net prior to release, post-slipping mortality rates can be unacceptably high. Atlantic mackerel (*Scomber scombrus*) support large and economically important purse seine fisheries but are known to be particularly vulnerable to such crowding-induced mortality. Developing management advice to promote post-slipping survival for this species is currently challenging, due to a lack of understanding of how crowding influences their physiology. Here we examine the physiological response, recovery and survival of wild caught mackerel exposed to various degrees and durations of simulated crowding stress in a series of sea cage trials. The magnitude of the physiological response and its time to recovery was positively correlated with crowding density and duration and was characterized by cortisol elevation, energy mobilization and anaerobic metabolite accumulation. There were also indications of osmoregulatory disturbance. Skin injury and mortality rates showed a similar positive relationship to crowding density. The physiological disturbance was recoverable for most fish. Instead, the rate at which mortalities developed and the physiological profile of moribund fish indicated that skin injury, likely arising from abrasive contact with netting and other fish during crowding, was the probable cause of mortality. Injured fish also exhibited a loss of allometric condition relative to non-injured survivors. Crowding treatments were potentially confounded by differences in ambient oxygen reduction, water temperature and pre-treatment fish condition between trials, and densities were replicated only once. These results contribute to the development of welfare conscious fishing practices that aim to reduce post-slipping mortality.

## Teaser

Mortality rates for released fish in Atlantic mackerel (*Scomber scombrus*) purse seine fisheries can be unacceptably high. By examining physiological responses and survival in simulated capture and release trials, we show that fish condition and survival rates may be enhanced by reducing crowding density and duration prior to release.

## Introduction

The capture of wild animals typically results in physiological disturbance (Iossa *et al.*, 2007; Cattet *et al.*, 2008; Cooke *et al.*, 2013a; Cook *et al.*, 2019b; Tylan *et al.*, 2020). The magnitude and duration of disturbance is largely determined by the intensity and duration of the capture stressors (Koolhaas *et al.*, 2011; Schreck and Tort, 2016), by the species and individual’s tolerance (so called intrinsic factors) and by environmental modifiers (extrinsic factors; Moberg (2000)). The same variables govern the likelihood of recovery to physiological stasis. The additional ‘allostatic load’ (the increased demand on biological systems due to stress; Ramsay and Woods, 2014) resulting from capture-induced physiological disturbance or injury can result in immediate or delayed mortality, or have important whole body sub-lethal effects that may affect population or ecosystem-level functions (Wilson *et al.*, 2014). When animals are retained for consumption, unresolved physiological disturbance may result in undesirable flesh quality alterations (Gregory, 1998). Understanding the physiological response of animals to capture is therefore fundamental for sustainable, profitable and rational natural resource utilization (Cooke *et al.*, 2013b).

In fish, perception of acute stressor events such as capture stimulates the release of catecholamine and corticosteroid hormones from the head kidney into the bloodstream (Wendelaar Bonga, 1997). The action of these hormones at their target organs mobilizes stored energy reserves and enhances oxygen availability for a fight, flight or coping response (Schreck and Tort, 2016). Under field conditions, researchers have typically quantified this response by blood sampling, often collected from the caudal vasculature (Sopinka *et al.*, 2016). Because they are endocrine mediated (Pottinger, 2007), physiological indicators may only begin to manifest sometime after the onset of stress (Lawrence *et al.*, 2020) and peak response may only be observed after some hours (Sopinka *et al.*, 2016). It is therefore important to quantify physiological disturbance after initial stressor exposure not only to accurately characterize recovery but also to encapsulate the full response. Sampling of moribund individuals may allow physiological status and mortality outcomes to be associated (e.g. Moyes *et al.*, 2006).

Good catch welfare practices may be defined as ‘capture and handling methods that minimise the physical damage to, and allostatic load on, any retained fish until after they are either slaughtered or released, and thus promote the likelihood for post-release survival and/or good product quality’ (Breen *et al.*, 2020a). In wild-capture commercial fishing, delayed mortality among unwanted and released catches is a significant challenge to the long-term sustainability of the industry (Davis, 2002). In northern European waters, small pelagic species that form large schools are targeted by purse seine fisheries. However, a lack of suitable hydro-acoustic technology means that individual catches in these fisheries routinely exceed the capacity or quota allocation of the vessels, leading to a need to release fish. Unwanted fish are released directly from the net while still in the water, in a process called ‘slipping’ (Marçalo *et al.*, 2019). Although slipped fish are typically alive when they leave the net, the potential for delayed mortality is high (Mitchell *et al.*, 2002; Huse and Vold, 2010; Tenningen *et al.*, 2012; Marçalo *et al.*, 2018). Excessive crowding is thought to be the main driver of mortality (Lockwood *et al.*, 1983; Marçalo *et al.*, 2006, 2010; Huse and Vold, 2010; Tenningen *et al.*, 2012). Regulations related to slipping in EU and Norwegian waters therefore attempt to minimize the severity and duration of crowding by stipulating fish release must take place before the net volume reaches a critical threshold (as determined by the length of net that has been hauled aboard: 87.5% in Norway, Lovdata, 2021; and 80% in some EU waters, EU, 2014a, 2014b). However, a lack of specific knowledge of how and to what extent crowding influences the physiology of small pelagic fish means that developing further regulations that could mitigate slipping mortality by reducing stressor induced allostatic loading is currently challenging.

Atlantic mackerel (*Scomber scombrus*) is an abundant, migratory and planktivorous small pelagic fish found in schools throughout coastal and continental shelf regions of the North Atlantic (Olafsdottir *et al.*, 2016). They support extensive fisheries throughout European waters, with annual landings in the region of 800 000 to 1 million tonnes (ICES, 2020). A substantial proportion of this catch is taken by purse seine (ICES, 2020), particularly in Norway where individual catches are typically in the region of 50–500 tonnes (Marçalo *et al.*, 2019) and where crowding is a fundamental part of the capture process. Mackerel are, however, highly vulnerable to crowding-induced delayed mortality. Large-scale field trials have indicated that post-slipping mortality rates can exceed 80% for this species (Huse and Vold, 2010). A series of smaller scale studies in the 1980s culminated in the conclusion that skin injuries arising from abrasive contact with the net and other fish was the major cause of mortality (Lockwood *et al.*, 1983). Physiological response and recovery were also examined in some of these studies (Pawson and Lockwood, 1980; Holeton *et al.*, 1982; Swift, 1983). Collectively, these studies provide physiological information on baselines, moribund fish and the response and recovery from various treatments (including simulated purse seine capture and release). It is, however, likely that these findings were compromised by highly stressful capture and transfer procedures. Furthermore, these early studies examined only a limited suite of physiological parameters and employed relatively short post-stressor monitoring times, which may have been inadequate to fully describe stressor related mortality (Breen and Catchpole, in press).

Mackerel are ectothermic and inhabit a non-stable environment (Schulte, 2014). Consequently, there is substantial potential for their physiological and survival response to stress to be modified by environmental factors. These particularly include temperature (by affecting the rate of metabolic reactions) and oxygen concentrations (by affecting metabolic capacity) (Barton, 2002; Schreck and Tort, 2016). Hypoxia may be an important compound stressor occurring during purse seine crowding (Tenningen *et al.*, 2012; Breen *et al.*, 2020b), arising due to limited water exchange through the dense biomass of crowded fish.

Stress and survival responses may also be modified by intrinsic factors (Schreck and Tort, 2016). Of particular relevance is condition, in terms of both allometric condition and physical condition. Allometric condition theoretically determines the available energetic capacity to cope with stressor induced increases in allostatic load (Moberg, 2000). Allometric change may also be a common whole body response in pelagic fish following interaction with fishing gear (e.g. Suuronen *et al.*, 1996; Marçalo *et al.*, 2010; Tenningen *et al.*, 2012). Reduced physical condition resulting from skin injuries are thought to be a major cause of death in slipped mackerel (Lockwood *et al.*, 1983) and other pelagic species (Misund and Beltestad, 1995; Mitchell *et al.*, 2002; Marçalo *et al.*, 2018). Such injuries may induce osmoregulatory challenges (Olsen *et al.*, 2012a; Cook *et al.*, 2019a) and provide a route for pathogenic infection (Sveen *et al.*, 2020).

The work presented in this manuscript was part of a larger project whose overarching objective was to develop indicators of stress to help define safe limits for the release of unwanted catches in commercial purse seining. Several different potential stress/welfare metrics (including behaviour, vitality, skin colour and meat quality) were examined. However, the primary objective of the current study was to describe the physiological and survival response of Atlantic mackerel to crowding stress (and thereby relate capture related stress to welfare outcomes) using a controlled, mesocosm-scale, experimental setup in sea-cages. A more comprehensive understanding of how mackerel physiologically respond to purse seine capture can contribute to the development of welfare conscious fishing practices that aim to promote post-release by minimizing allostatic loading (Breen *et al.*, 2020a). We simulated various degrees and durations of crowding in a series of eight trials. Specifically, we aimed to (i) determine physiological responses during crowding; (ii) determine responses and survival rates following the cessation of crowding (analogous to slipped fish in the fishery); (iii) examine the relationship between different degrees of stress, physiological responses and mortality; and (iv) monitor intrinsic and extrinsic factors that may modify this relationship. By employing a recently developed husbandry technique (involving the passive transfer of substantial numbers of wild-caught mackerel into aquaculture net cages) with extended post-stressor monitoring periods, we aimed to overcome the limitations of previous studies. We also examine a wider range of parameters to gain a more holistic understanding of any physiological disturbance. Although large-scale field trials (e.g. Huse and Vold, 2010; Tenningen *et al.*, 2012) may be more representative of commercial crowding conditions and therefore allow extrapolation of survival rates for the fishery, our primary aim was to characterize how mackerel respond to and recover from crowding. As such, mesocosm-scale experiments such as described here give a better opportunity to examine stressors and responses at an affordable cost, while still being more representative of field conditions than laboratory studies.

## Materials and methods

### Fish capture and husbandry

Feed pellets were used to attract wild adult mackerel into aquaculture net cages at the Austevoll Research Facility (60°N) of the Institute of Marine Research, Norway, in the summers of 2018 and 2019. Fish were housed in a 1728-m^3^ ‘holding cage’ mounted on a floating pontoon for either ~10 months (fish collected in 2018 and used in the May/June trials) or ~1.5 months (collected in 2019 and used in the August/September trials) (Table 1). The pontoon was anchored ~100 m offshore (water depth: ~ 45 m) and could be accessed on foot from the shore by a bridge. Fish foraged on natural prey items with the occasional addition of feed pellets.

**Table 1:** Summary data of various trials investigating the effect of crowding on Atlantic mackerel physiology and survival (various applied crowding densities and their durations are indicated)

Period	Trial name	Dates (from pre-treatment to trial end)	Survival monitored?	Treatment duration (decimal hours)	Total trial duration (decimal hours)	Estimated density pre-treatment density (kg/m^3^)	Estimated during- treatment density (kg/m^3^)	Cage volume during treatment (m^3^)
May/June	Control 1	21 May 2019–21 May 2019	No	1.87	1.87	0.76	0.76	149.17
	Control 2	28 May 2019–6 June 2019	Yes	0.68	219.9	0.61	0.59	149.17
	High and prolonged 1	22 May 2019–22 May 2019	No	1.13	1.13	0.41	data missing	data missing
	Low	29 May 2019–6 June 2019	Yes	0.25	199.35	1.19	92.00	1.88
	High and prolonged 2	6 June 2019–6 June 2019	No	1.15	1.15	0.45	182.75	0.37
August/September	Control 3	21 August 2019–17 September 2019	Yes	0.75	648	0.78	0.76	149.17
	Moderate	22 August 2019–11 September 2019	Yes	0.22	481	0.59	146.21	0.58
	High	28 August 2019–17 September 2019	Yes	0.25	481	0.64	179.87	0.51

A minimum of 48 hours before beginning the individual trials, ~100 fish each (estimated visually) were transferred from the holding cage into two smaller experimental cages (dimensions: 5 × 5 × 5 m with an inverted 2.9-m-deep pyramidal section at the bottom; mesh size: 36 mm; total volume: 149.17m^3^). Experimental cages were situated inside the holding cage. Transfer was achieved with minimal disturbance by using feed pellets to encourage fish to enter voluntarily. The almost immediate adoption of normal schooling and feeding behaviours indicated rapid adaption to the experimental cages. The lack of transfer mortalities and skin injuries in pre-treatment samples further indicated transfer procedures were benign.

### Ethics statement

All experimental protocols were prospectively authorized by the Norwegian animal welfare authority (Mattilsynet, FOTS licence ID: 19238). Experimental design considered the 3R’s. There was no alternative to the use of live animals. Numbers used were the minimum required to (i) achieve practical and realistic crowding densities, (ii) precisely estimate survival while accounting for removals for sampling (as determined by binomial power analysis) and (iii) provide adequate school sizes for behavioural enrichment and quantification (results not reported here). No anaesthesia was used so as not to compromise experimental objectives. All fish were euthanized using a percussive blow to the head prior to any invasive sampling.

### Crowding treatments

A total of eight trials of various crowding densities and durations were conducted in either May/June or August/September 2019 (Table 1). Crowding was achieved by manually lifting cages to reduce their in-water volume, thereby gathering fish in the lower pyramidal section. This procedure typically took ~5 minutes. There is currently no empirical data from the fishery of typical crowding densities (Tenningen *et al.*, 2015). Therefore, the applied densities were intended to (i) exceed previously investigated crowding levels in which no mortalities were induced (~88 kg/m^3^, Handegard *et al.*, 2017), (ii) exceed modelling-derived estimates of density for the early stages of capture in the fishery (Tenningen *et al.*, 2019) and (iii) inhibit normal schooling behaviour while ensuring the majority of fish were not air exposed. In this way, we expected to induce mortalities using a range of densities somewhat representative of the final stages of a real capture event (i.e. up to and beyond the Norwegian release limit of 87.5% of the net length).

Three trials (‘Low’, ‘Moderate’ and ‘High’) investigated physiological response and survival by crowding schools for ~15 minutes and then allowing the cage to sink back to maximum volume under its own weight (analogous to a typical, regulation compliant, crowding and slipping event in the fishery; Anders *et al.*, 2019a). These trials were monitored for post-crowding survival for either 8 or 20 days (Table 1).

Two further trials (‘High and prolonged 1’ and ‘High and prolonged 2’; Table 1) crowded fish for ~ 60 minutes (analogous to the typical time for non-slipped, large retained catches to be pumped from net and onto the catching vessel in the fishery) to determine the effect of more extreme stress on physiology. No survival monitoring was undertaken for the high and prolonged trials. Instead, these trials were terminated immediately after treatment, because we and the Norwegian animal welfare authority (Mattilsynet) agreed that the severity of this treatment was very high and would likely induce a high mortality and unacceptable levels of post-treatment stress.

Captivity effects were controlled for with three control trials (Table 1) during which cage volume was not altered. ‘Control 1’ acted as the control for the two high and prolonged trials. The ‘Control 1’ group of fish subsequently acted as the control (‘Control 2’) for the ‘Low’ survival trial. At the completion of ‘Control 2’, the same group was re-used again as the ‘High and rolonged 2’ trial. This was considered acceptable as they had received no crowding treatment prior to this point and had exhibited 100% survival throughout. ‘Control 3’ acted as the control for the ‘Moderate’ and ‘High’ trials in August/September.

We estimated crowding densities (Table 1) by considering the biomass of fish (calculated from individual fish weights collected during sampling and at the end of trials) and an estimate of cage volume during treatment. For volume estimates, we first estimated net area at the surface using five images collected by an overhead video camera during treatment (analysed in ImageJ V1.51 software; Schneider *et al.* (2012)). Assuming the cage bottom formed a right cone shape during crowding, volume was estimated as $V=(A\times D)/3$, where *A* is the area of the base cone (i.e. area at the surface) and *D* is the geometrically derived water depth. Theoretical control densities were calculated using the unrestricted volume of the cage (149.17m^3^), assuming the fish adopted a density dependent on cage volume. In reality, uncrowded captive mackerel tend to adopt a higher voluntary density than the available space defined by the theoretical crowding density (Handegard *et al.*, 2017).

### Monitoring physiological responses

For the ‘Low’, ‘Moderate’ and ‘High’ trials and their controls (‘Control 2’ and ‘Control 3’), we attempted to sample five fish for physiology at the following time periods: (i) within <30 minutes prior to commencing treatment (‘pre-treatment’); (ii) ~2 hours after the end of treatment (‘2 hours post-treatment’); (iii) ~24 hours after the end of treatment (‘24 hours post-treatment’); and (iv) within <2 hours prior to the termination of the trial (‘termination’). During treatment, we sampled as many fish as possible within the ~15-minute crowding period [mean number of fish sampled ±95% confidence interval (CI): 8 ± 1.13]. During the corresponding period for the control trials, we sampled 10 fish.

Due to logistical and fish welfare reasons, there was no pre- or post-treatment sampling for the ‘high and prolonged’ trials or their control (‘Control 1’). For these extreme trials, we sampled as many fish as possible within the first 15 minutes. Following this, we sampled three additional fish every ~10 minutes. Finally, five fish were sampled at the end of the ~60-minute crowding period, resulting in a mean sample size of 22 ± 2.94 fish. The sample size for ‘Control 1’ was 21 fish.

Fish were collected from the cages using one of two different methods, depending on sampling period. All control trial samples were collected by first scattering feed pellets to encourage feeding behaviour. Fish were then individually caught using barbless hooks (size: #1/0) on handlines and immediately removed from the water. The same method was used to collect pre- and post-treatment samples for crowding trials. Capture by hook did not appear to unduly disturb the remaining fish in the cage that continued to feed and did not show pronounced avoidance responses. During treatment for crowding trials, it would not have been possible to collect fish using the handline method due to the inhibition of appetite under stress (Conde-Sieira *et al.*, 2018). Consequently, fish were randomly collected using a dip net. Any increase in stress due to the use of the dip net can be expected to be minor in comparison to that incurred by the crowding treatment itself. Moreover, any additional stress due to the collection method would be unlikely to appear as a signal in the physiological response because of the short time period (<3 minutes) between collection and blood sampling (see below).

After collection, all fish were first assessed for behavioural vitality while handling, because this could be unobtrusively incorporated into the euthanization procedure. Alternate fish were also assessed for vitality in a 70-l bucket of seawater prior to the during-handling assessment (results not reported here). The in-water vitality assessment procedure did not significantly influence any of the blood physiology parameters (*P* ≥ 0.05 in all cases, Generalized Least Squares modelling in combination with Wald *F* testing) and the two groups were consequently combined in any subsequent statistical analysis.

The following sequential protocol was then applied to each fish: (i) euthanization out of water using a percussive blow to the head, (ii) photographed to assess stress related colour change (results not reported here), (iii) total weight recorded, (iv) 1.5–3 ml of blood collected from the caudal vasculature using 5-ml syringes with 21G needles and 10% EDTA (ethylenediaminetetraacetic acid) anticoagulant, (v) muscle pH determined from a scalpel incised opening in the dorsal side of the left fillet immediately posterior to the gill operculum (results not reported here) and (vi) fork length recorded (measured to the nearest cm below). The mean (± 95% CI) time between collection of fish and completing blood sampling was 1.07 (± 0.17) minutes (range: <1–5 minutes), with 97% being completed within best practice guidelines of 3 minutes (Lawrence *et al*., 2020). The blood sample was used to quantify the physiological responses presented in this study. Collected blood was immediately chilled on ice for no more than ~4 hours for later processing. Blood was analysed for whole blood haematocrit (HCT) and plasma pH, cortisol, potassium ions (K^+^), chloride ions (Cl^−^), sodium ions (Na^+^), osmolality, glucose and lactate concentrations using the same equipment and methodology as described in Anders *et al.* (2020).

### Monitoring survival outcomes

Trials in which survival was monitored (Table 1) were monitored up to twice daily (morning and afternoon, ~6 hours apart). The conical base of the cage facilitated the collection of sunken moribund or dead fish into a net ‘collection device’ positioned in the centre. By means of ropes, the collection device could be raised to the surface with minimal disturbance to surviving fish. Moribund fish were identified by (i) immobility and weak response to tactile stimuli or (ii) sustained atypical behaviour (typically, failure to school). Such fish were collected at the surface using a dip net (which healthy mackerel easily avoid). Moribund fish were physiologically sampled using the same protocol described above. The monitoring period continued until it was clear that mortality rates had reached asymptote (Breen and Catchpole, *in press*). For trials in which no mortality was recorded, monitoring periods continued until it was evident mortalities were unlikely to occur. All surviving fish were euthanized at the end of trials by a percussive blow or MS-222 anaesthetic overdose. Fork length and total weight was then recorded.

### Monitoring potential extrinsic and intrinsic modifiers

We monitored environmental conditions during trials using a SAIV CTD (Model: SD204) fitted with a RINKO III electronic oxygen sensor (JFE Advantach Co., Ltd). The instrument was placed in the approximate centre of the cages at a depth of ~3.5 m using a pulley system. During crowding treatments, the instrument was placed in the approximate centre of the crowded school.

To understand if condition indices influenced physiological and survival responses in mackerel, we calculated Fulton’s condition factor (K = 100 × [total weight/fork length^3^], where weight is expressed in g and length in cm) and noted the presence or absence of skin injuries for trials in which survival was monitored (Table 1). An insult to the skin of any type, severity or location that was found during visual inspection was sufficient for a fish to be considered as injured. It was not feasible to examine the effects of condition in the high and prolonged trials (Table 1), due to the lack of post-treatment monitoring and the latency in which mackerel skin injuries develop after crowding (Lockwood *et al.*, 1983; Handegard *et al.*, 2017).

### Statistical analysis

For trials in which we monitored post-treatment survival (Table 1), we modelled survival as a function of ‘time’ (continuous: time since treatment start in decimal hours) and ‘trial’ (categorical: ‘Low’, ‘Moderate’, ‘High’, ‘Control 2’, ‘Control 3’) using the non-parametric Kaplan–Meier estimator (Rich *et al.*, 2010) from the ‘survival’ R package (Therneau, 2015). We also fit individual models to each trial in which mortalities occurred, to determine if skin injuries (Bernoulli: injured or not) affected survival. We incorporated right censored observations corresponding to survivors or physiological sampling removals. Time of death for dead or moribund fish was considered to occur at the point of removal. Differences in survivorship were tested using a log-rank test.

We employed generalized least squares (GLS) models in the ‘nlme’ R package (Pinheiro *et al.*, 2019) to examine physiological response and recovery. GLS modelling allows the incorporation of residual dependency into models via variance functions and takes the form of simple linear regression when variance functions are not applied (Zuur *et al.*, 2009). Cortisol samples above the limit of quantification (*n* = 20) for the analytical machine were assigned the manufacturer quoted upper limit (800 ng/ml). We fitted separate interactive and main effects models for each physiological parameter, containing the variables ‘trial’ (categorical: ‘Low’, ‘Moderate’, ‘High’, ‘Control 2’ or ‘Control 3’; i.e. only trials in which survival was monitored) and ‘monitoring period’ (categorical: ‘pre-treatment’, ‘treatment’, ‘2 hours post-treatment’, ‘24 hours post-treatment’ or ‘termination’).

To investigate the time course of physiological response during periods when fish were crowded, we fitted separate interaction and main effects GLS models for each physiological parameter. These considered the variables of ‘trial’ (categorical: ‘Low’, ‘Moderate’, ‘High’, ‘High and prolonged 1’, ‘High and prolonged 2’, ‘Control 1’, ‘Control 2’ or ‘Control 3’) and ‘exposure time’ (time since treatment start, transformed via ln (1 + X) to account for any non-linear time effect). Treatment start was considered to be when the first fish was removed from the cage for sampling. Temporal correlation structures were excluded from final models if they did not significantly improve the fit (Zuur *et al.*, 2009). To prevent non-sensical negative predictions for the cortisol datasets, we modelled this parameter using generalized linear model (GLM) with a gamma error distribution and a log link function. GLS models were also used to test for differences in oxygen levels during crowding between trials and their control, as well as within trials to compare pre- and during-treatment periods. The significance of terms in GLS and gamma GLM models was determined using Wald *F* testing.

To test for any differences in ‘fork length’ and ‘condition factor’ (both continuous), we fit a generalized linear mixed model (GLMM) (Zuur *et al.*, 2009) to a dataset comprised of fish sampled prior to or during treatment (to exclude any possibility of treatment effects). This model consisted of the fixed variable ‘trial’ (categorical: ‘Low’, ‘Moderate’, ‘High’, ‘High and prolonged 1’, ‘High and prolonged 2’, ‘Control 1’, ‘Control 2’ or ‘Control 3’) and random effects to account for the lack of independence between trials in which we re-used the same group of fish. A GLS was fit to investigate potential responses in condition factor after crowding. For this, we considered variables of ‘status’[categorical: (i) pre- and during-treatment samples, (ii) injured survivors, (iii) non-injured survivors, (iv) moribund fish and (v) dead individuals] and ‘trial’ (categorical: ‘Low’, ‘Moderate’, ‘High’, ‘Control 2’ or ‘Control 3’; i.e. only trials in which survival monitored). One erroneous outlier in condition was identified by Cleveland dot plots and removed. Likelihood ratio testing (LRT) was used to determine significance in GLMMs.

We examined how the probability of post-crowding injuries varied between trials, using a binomial GLM with a logit link function. Quasibinomial GLMs (to account for overdispersion) were used to investigate the relationship between ‘density’ (continuous: crowding density during treatment) and rates of mortality and injury. LRT was used to determine significance in binomial GLM’s.

All statistical analysis was undertaken using R version 3.4.2 (R Core Team, 2017). All modelling procedures started with maximal models (considering all appropriate interactions and variance structures) and were reduced, where appropriate, to the most parsimonious through significance and AIC testing. Model fits were assessed visually using residual plots (Zuur *et al.*, 2009).

## Results

A total of 934 mackerel were used throughout the different trials (Table 2). The mean (± 95% CI) number used per trial was 143 ± 34.81 (Table 2). Overall mean fork length and total weight for fish sampled during the pre- and during-treatment periods was 38.36 ± 0.37 cm and 738 ± 24 g, respectively. Fish length did not differ significantly between trials (df = 7, LRT = 5.64, *P* = 0.582).

**Table 2:** Numbers of mackerel used in trials investigating the effect of crowding on Atlantic mackerel physiology and survival (survival, mortality and injury rates are included)

Trial name	No. of fish prior to pre-treatment	No. of fish exposed to treatment	No. of survivors^a^	Proportion of injured survivors	No. of mortalities (excl. moribund fish)	No. of moribund fish	Mortality proportion (95% CI^b^)
Control 1	149	149	NA	NA	NA	NA	NA
Control 2	121	116	96	0.010	0	0	0.000 (0.000, 0.038)
High and prolonged 1	78	78	NA	NA	NA	NA	NA
Low	236	231	213	0.169	0	0	0.000 (0.000, 0.018)
High and prolonged 2	91	91	NA	NA	NA	NA	NA
Control 3	180	175	155	0.013	0	0	0.000 (0.000, 0.024)
Moderate	136	131	112	0.420	3	0	0.026 (0.009, 0.074)
High	155	150	91	0.648	32	8	0.305 (0.233, 0.389)

^a^Removals for physiological sampling immediately prior to termination (‘Termination’) were classed as survivors.

^b^CIs were calculated using Wilson score intervals.

### Environmental conditions

During-treatment water temperatures for trials conducted in August/September were broadly similar, as were trials conducted in May/June (Table 3). However, overall water temperatures were ~4.5°C warmer for the August/September trials compared to the May/June trials (Table 3). Relative to pre-treatment levels, crowding treatments were associated with significant reductions (*P* < 0.001 in all cases) in ambient dissolved oxygen concentrations not observed during control trials (typically a 1–2 mg/l reduction; Table 3). During crowding, oxygen levels were significantly lower than for the corresponding period in the respective control trial (*P* < 0.001 in all cases, Table 3). Oxygen minimums were particularly pronounced for the moderate and high crowding trials in August/September (Table 3). It is noteworthy that during the May/June there was a phytoplankton bloom at the trial site that resulted in supersaturated dissolved oxygen in surface waters (mean ± 95% CI: 112 ± 4.25%).

**Table 3:** Environmental conditions during trials investigating the effect of crowding on Atlantic mackerel physiology and survival (values indicate means ± 95% CIs unless otherwise indicated)

			Pre-treatment	During treatment		
Period	Trial name	Treatment date	Oxygen concentration (mg/l)	Temperature (°C)	Oxygen concentration (mg/l)	Oxygen minimum (mg/l)
May/June	Control 1	21 May 2019	10.096 ± 0.007	11.608 ± 0.007	10.247 ± 0.004	9.26
	Control 2	28 May 2019	9.893 ± 0.002^a^	10.991 ± 0.003	data missing	9.81
	High and prolonged 1	22 May 2019	10.952 ± 0.003^a^	12.257 ± 0.009	9.339 ± 0.012^b^	8.71
	Low	29 May 2019	9.877 ± 0.002	data missing	data missing	data missing
	High and prolonged 2	6 June 2019	9.616 ± 0.001	13.880 ± 0.024	9.080 ± 0.016^b^	7.39
August/September	Control 3	21 August 2019	8.356 ± 0.002	16.425 ± 0.001	8.386 ± 0.001	8.37
	Moderate	22 August 2019	7.863 ± 0.005	16.067 ± 0.002	6.182 ± 0.014^b^	5.21
	High	28 August 2019	8.241 ± 0.007	17.883 ± 0.002	6.781 ± 0.028^b^	5.22

§ ^a^Measured <1.5 hours after treatment period.

^b^Significantly different (*P* < 0.05, GLS modelling with Wald *F* testing) during treatment from both pre-treatment levels and the corresponding control trial.

### Survival responses

**Figure 1:**
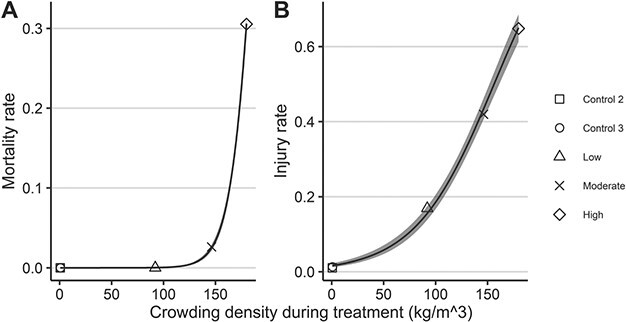
The relationship between crowding density during crowding trials and (**A**) mortality rates and (**B**) injury rates in Atlantic mackerel. Injury rates are among survivors remaining at the end of the trials. Shaded areas indicate model derived 95% CIs, with the underlying dataset indicated by different shaped points according to trial.

The probability of survival was significantly different between the trials (log rank test: df = 4, *χ*^2^ = 139, *P* < 0.001). There were no dead or moribund fish from the ‘Low’ crowding trial (conducted in May/June) or its control (‘Control 2’; Table 2). No mortalities occurred in the ‘Control 3’ trial during August/September. However, ‘Moderate’ and ‘High’ crowding induced overall mortalities of ~3% and ~30%, respectively (Table 2). The positive relationship between mortality rates and crowding density during treatment was highly significant (Fig. 1; LRT = 135.36, df = 1, *P* < 0.001).

**Figure 2:**
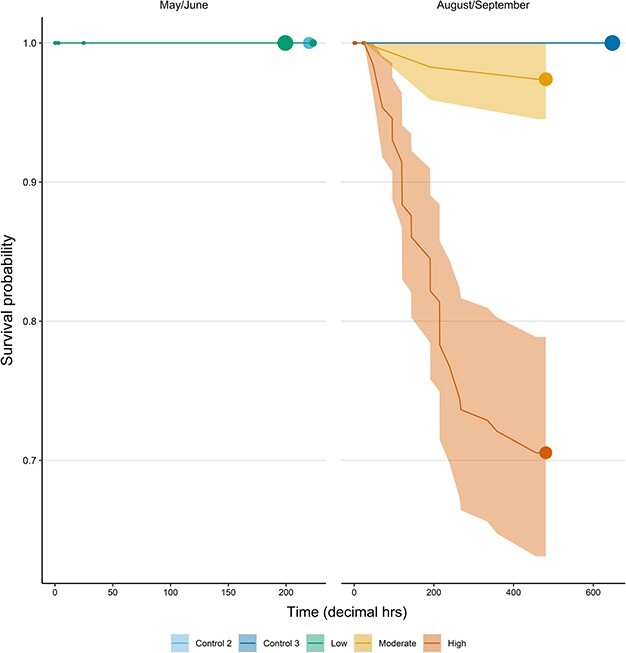
Survival responses of Atlantic mackerel to crowding in simulated trials. The fitted regressions describe the mean probability (with 95% CIs as shaded areas) of survival for the various trials, derived from the Kaplan–Meier survival function. Circles indicate the occurrence of right censored observations and are scaled in size within each plot according to the number of observations.

All mortalities (total throughout all trials: 35) and moribund fish (total: 8) were collected during the morning, suggesting they occurred sometime during the night. Mortalities in the moderately crowded trial first occurred ~8 days (191 hours) post-crowding, with a further single event at ~19 days (460 hours; Fig. 2). Mortalities in the highly crowded trial occurred more rapidly and consistently (Fig. 2). The first was recorded ~2 days (47 hours) post-treatment and continuing at a mean rate (± 95% C.I) of 3.6 ± 1.06 per day (range: 1–6 fish per day) until ~11 days (264 hours) post-crowding. After this, mortality rates decreased to 0.4 ± 0.43 fish per d, with a further total of 4 events at 14 days (335 hours), 15 days (358 hours) and 19 days (456 hours) post-treatment (Fig. 2).

### Skin injuries

**Figure 3:**
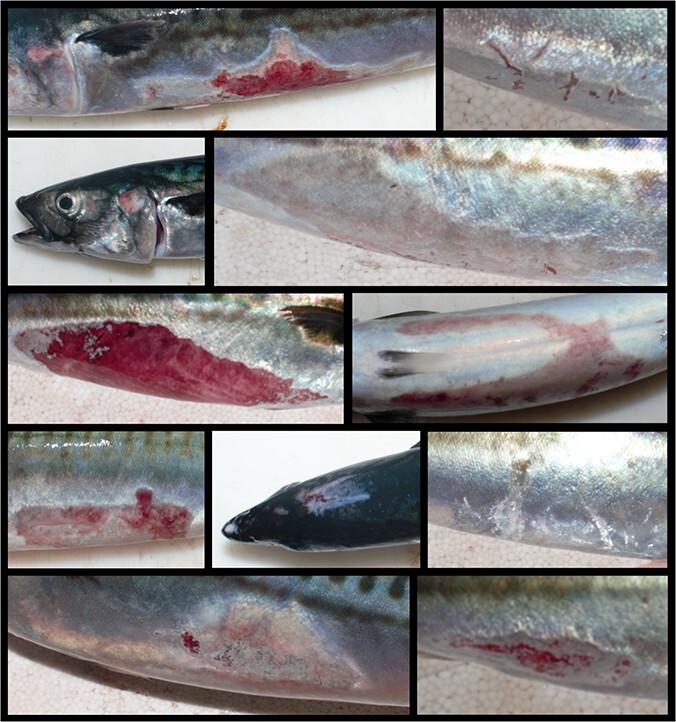
Examples of post-crowding skin injuries in Atlantic mackerel.

No fish collected prior to or during treatment were injured. However, substantial numbers of surviving fish were classified as injured at the end of trials in which survival was monitored (Table 2). Injuries ranged from superficial damage and scale loss, through to ulcerated exposure of the underlying dermis and muscle tissue (Fig. 3). The probability of injury differed significantly between trials (LRT = 275.97, df = 4, *P* < 0.001), with higher crowding densities increasing injury rates (Fig. 1, LRT = 201.3, df = 1, *P* < 0.001). All moribund or dead fish were injured. Consequently, injuries were associated with a marginally (log rank test: df = 1, *χ*^2^ = 4, *P* = 0.05) or highly significant (df = 1, *χ*^2^ = 15.8, *P* < 0.001) decrease in survival probability in the ‘Moderate’ and ‘High’ crowding trials respectively.

**Figure 4:**
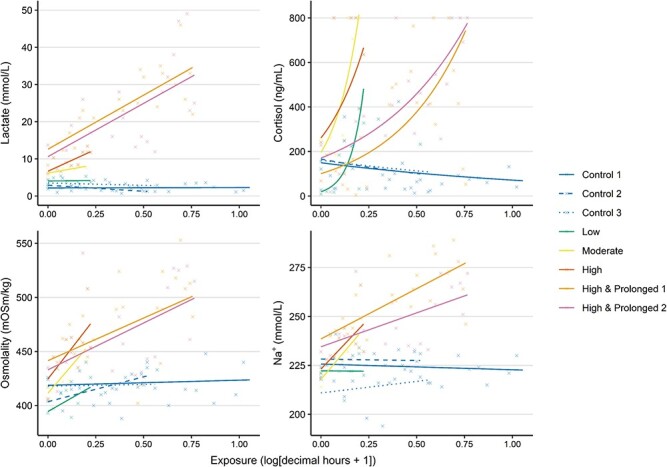
The development of selected physiological parameters of Atlantic mackerel during the various crowding trials. The fitted regressions describe the interactive relationship between crowding exposure time and trial. The underlying dataset is indicated as crosses.

**Table 4:** Statistical test results and associated significance for various blood physiology parameters during crowding of Atlantic mackerel

			Main effects models						Interactive models			
			X_1_ (‘trial’)			X_2_ (‘exposure’)			X_3_ (X_1_ × X_2_ interaction)			
Parameter	*n*	Variance structure	df	*F*-value	*P*	df	*F*-value	*P*	Variance structure	df	*F*-value	*P*
Cortisol^a^	103	NA	7	8.0683	<0.001	1	4.3130	0.041	NA	7	4.283	<0.001
Lactate	103	VarIdent (trial)	7	58.3791	<0.001	1	0.0694	0.793	VarIdent (trial)	7	9.598	<0.001
Haematocrit	103	None	7	5.060	<0.001		2.123	0.148	None	7	0.830	0.565
pH	103	corSpher	7	3.69	<0.001	1	0.06	0.807	corSpher	7	0.410	0.896
Osmolality	103	VarIdent (trial)	7	15.11	<0.001	1	7.44	0.008	VarIdent (trial)	7	3.450	0.003
Glucose	103	VarIdent (trial)	7	9.555	<0.001	1	1.979	0.163	VarIdent (trial)	7	0.565	0.782
Potassium ions	103	corSpher VarIdent (trial)	7	7.0501	<0.001		0.8985	0.346	corSpher VarIdent (trial)	7	0.802	0.588
Sodium ions	103	None	7	24.04	<0.001	1	18.24	<0.001	None	7	3.730	0.001
Chloride ions	103	VarIdent (trial)	7	10.956	<0.001	1	0.837	0.363	VarIdent (trial)	7	1.578	0.153

§ ^a^Data modelled using GLM with gamma error distribution and log link function. Both main and interactive effects of ‘trial’ and ‘exposure’ (time since treatment start) were fit using GLS models (unless otherwise indicated) using a variety of variance structures.

**Table 5:** Statistical test results and associated significance for various blood physiology parameters throughout crowding trials on Atlantic mackerel

			Main effects models						Interactive models			
			X_1_ (‘trial’)			X_2_ (‘monitoring period’)			X_3_ (X_1_ × X_2_ interaction)			
Parameter	n	Variance structure	df	*F*-value	*P*	df	*F*-value	*P*	Variance structure	df	*F*-value	*P*
Cortisol^a^	143	NA	4	12.707	<0.001	4	2.555	0.041	NA	16	2.458	0.003
Lactate	143	VarIdent (trial × monitoring period)	4	85.935	<0.001	4	12.666	<0.001	VarIdent (trial × monitoring period)	16	10.948	<0.001
Haematocrit	143	None	4	4.851	0.001	4	1.752	0.142	None	16	1.393	0.157
pH	143	VarIdent (monitoring period)	4	1.740	0.145	4	8.360	<0.001	VarIdent (monitoring period)	16	2.720	0.001
Osmolality	142	VarIdent (trial)	4	6.670	<0.001	4	10.380	<0.001	VarIdent (monitoring period)	16	3.570	<0.001
Glucose	143	VarIdent (monitoring period)	4	3.049	0.019	4	14.471	<0.001	VarIdent (monitoring period)	16	5.367	<0.001
Potassium ions	143	VarIdent (trial)	4	5.430	<0.001	4	4.202	0.003	None	16	3.447	<0.001
Sodium ions	143	VarIdent (monitoring period)	4	3.040	0.0196	4	19.49	<0.001	None	16	2.870	<0.001
Chloride ions	143	VarIdent (trial)	4	13.400	<0.001	4	9.260	<0.001	VarIdent (trial)	16	4.390	<0.001

§ ^a^Data modelled using GLM with gamma error distribution and log link function. Both main and interactive effects of ‘trial’ and ‘monitoring perio’ were fit using GLS models (unless otherwise indicated) using a variety of variance structures.

### Development of physiological responses during crowding

Upon crowding, some parameters responded rapidly and substantially: cortisol, lactate, osmolality and sodium (Na^+^) ions (Fig. 4). For these parameters, higher rates of increase were generally associated with the higher density treatments, with comparatively stable control observations (Fig. 4). This is supported by significant interaction terms in the GLS models (Table 4). Rates in the ‘Low’ crowding treatment typically mirrored those of the control trials for these parameters, except for cortisol where rapid change similar to the higher density trials was recorded (Fig. 4). For the high and prolonged trials, higher levels of disturbance at maximum exposure time were recorded for all four parameters apart from cortisol (Fig. 4), which was censored at 800 ng/ml by the limits of the analytical machine. Outside of these significant interactive effects, trends were less evident. Models often predicted contrary positive and negative relationships between trials and exposure time as a main effect was generally a poor predictor of physiological outcomes (Table 4).

### Physiological responses during and after crowding

**Figure 5:**
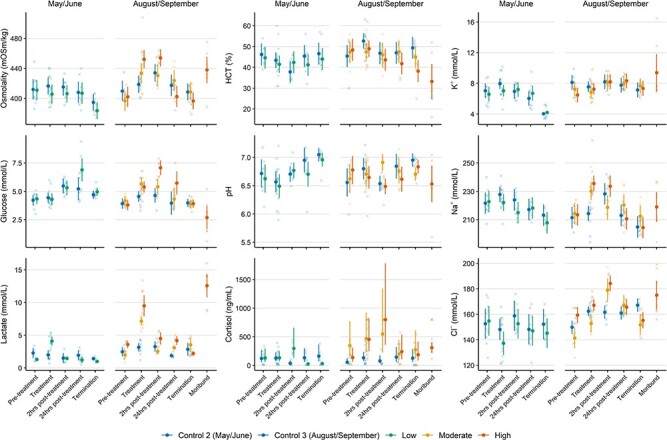
The physiological response to crowding and subsequent recovery for Atlantic mackerel during trials in which survival was monitored. Points and whiskers indicate the model derived mean (± 95% CIs) response, describing the interactive relationship between monitoring period and trial. The underlying dataset is indicated as crosses. To provide context, physiological profiles for moribund individuals are also presented. Estimates and confidence intervals for moribund individuals were derived directly from the data.

For trials in which physiology was monitored both during and after crowding, significant interactive effects of ‘trial’ and ‘monitoring period’ were found for all parameters apart from HCT (Table 5). The magnitude of increase in lactate during crowding mirrored the density differences between trials, and were 2.07 (low crowding), 2.27 (moderate crowding) and 3.03 times (high crowding) more than their respective controls (Fig. 5). Lactate recovery also depended on crowding density. Fish in the low and moderate crowding trials appeared to recover by 2 hours post-treatment, while highly crowded fish displayed elevated values at 24 hours (Fig. 5). Consequently, the interactive effect of ‘monitoring period’ and ‘density’ on lactate was highly significant (LRT = 10.95, df = 16, *P* < 0.001).

The development of other parameters also depended on crowding density. Responses during treatment for cortisol or glucose was evident in the ‘Moderate’ and ‘High’ trials, but not in the ‘Low’ trial (Fig. 5). Cortisol and glucose were also clearly elevated post-crowding (Fig. 5). However, while cortisol levels had generally recovered to baseline by 24 hours post-treatment, glucose disturbance was evident up to 24 h post-treatment in the ‘Low’ and ‘High’ trials (Fig. 5). Cl^−^ showed no crowding attributable change during the ‘Low’ trial, but was notably higher than control levels by 2 hours post-treatment (with recovery by 24 hours) in the ‘Moderate’ and ‘High’ trials (Fig. 5). A somewhat similar trend was evident for sodium for the ‘High’ crowding trial. Otherwise, there were few indications that changes to plasma pH, K^+^ or HCT were in response to crowding (Fig. 5).

The physiological profile of moribund fish was variable but notably disturbed (Fig. 5). In general, elevated levels of lactate, plasma ions (particularly K^+^) and osmolality with depressed levels of glucose were observed. Lactate and glucose values were among the most extreme observed during any of the trials (Fig. 5). HCT values were generally low and variable (Fig. 5).

### Allometric condition

**Figure 6:**
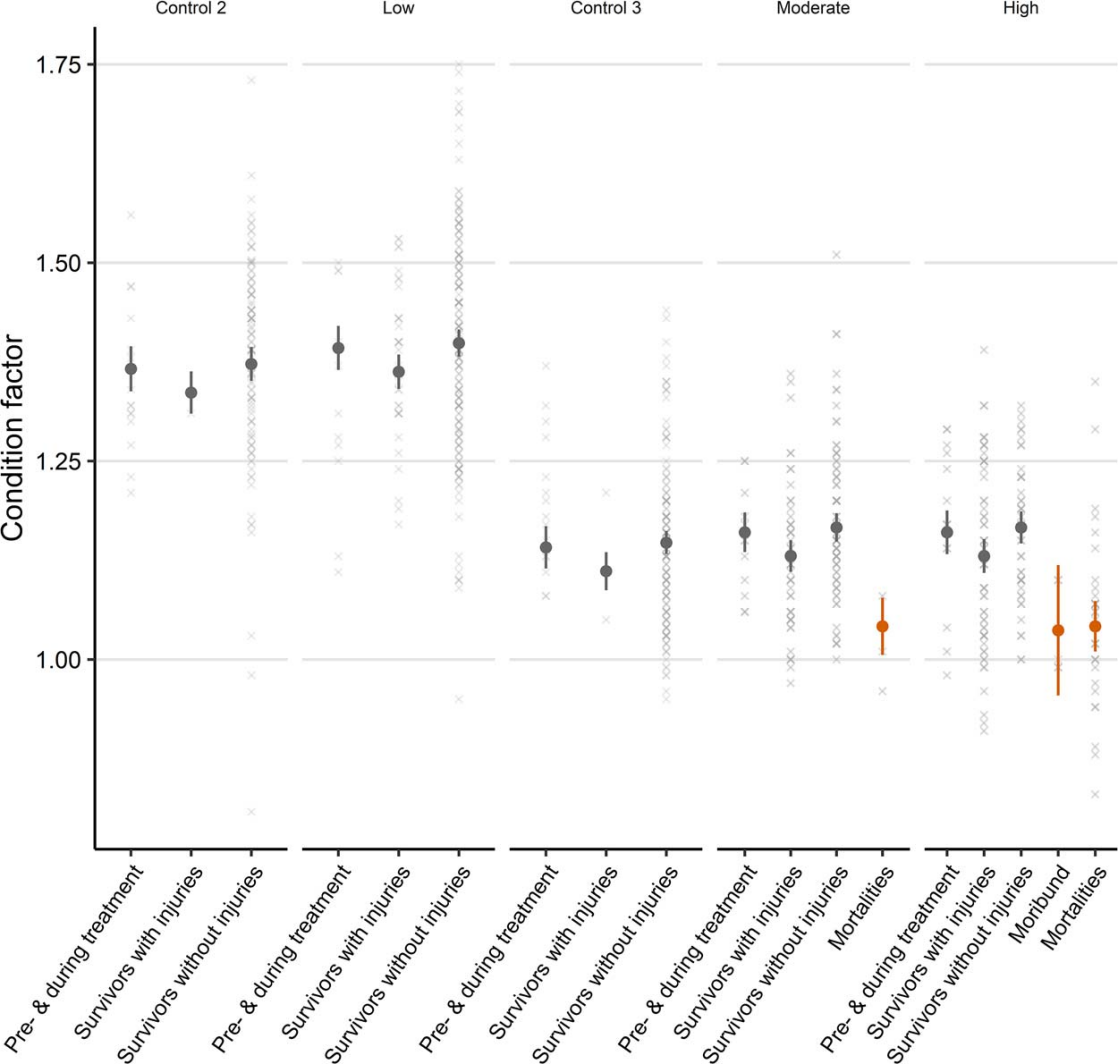
The effect of Atlantic mackerel skin injury on post-treatment condition factors (calculated as 100 × [total weight/fork length^3^]) during crowding trials in which survival was monitored. Pre- and during-treatment condition, as well as moribund fish and mortalities, are included for comparison. Points and whiskers represent model derived mean and 95% CIs, with the underlying dataset indicated as crosses.

The overall mean (± 95% C.I) allometric condition of fish sampled during the pre- and during-treatment periods was 1.30 ± 0.02100 g.cm^−3^. However, condition varied significantly between trials (df = 7, *F* = 11.274, *P* < 0.001), with the fish used in August/September having a notably lower condition than for the May/June trials (Fig. 6; 1.17 ± 0.03 vs 1.35 ± 0.03100 g.cm^−3^, respectively).

In the post-treatment period, the presence of injuries affected condition significantly (df = 4, LRT = 38.66, *P* < 0.001). The condition of injured survivors was lower than for non-injured survivors (Fig. 6, reduction in mean condition: 0.04100 g.cm^−3^; 3% reduction). Moribund and dead fish condition was similar to one another but was lower than for injured survivors (by a mean of 0.09100 g.cm^−3^; 7%, Fig. 6). Condition between non-injured survivors and pre- and during-treatment individuals was similar (Fig. 6).

## Discussion

The lack of pre-treatment and control trial mortalities suggests our capture and husbandry methods were benign. By minimizing the influence of sampling and captivity effects on physiological and survival responses, and by examining a wider suite of physiological parameters over longer post-stressor monitoring periods, our results extend the findings of previous authors (Pawson and Lockwood, 1980; Holeton *et al.*, 1982; Swift, 1983).

### Responses to crowding

The elevated cortisol results indicate that mackerel generally perceived crowding as a stressor (Ellis *et al.*, 2012). The physiological response to the stressor involved energy production from anaerobic pathways (marked by elevated lactate), which may have been exacerbated by the observed reduction in ambient oxygen during some trials. Concurrent and subsequent energy mobilization (hyperglycemia) and osmoregulatory disturbance (elevated osmolality, sodium and chloride levels) was also involved. Not considering the moribund fish and mortalities, physiological change was transient with recovery to baselines for most parameters by 24 hours and certainly by the end of trials. This physiological response is consistent with previous stress studies on mackerel (Pawson and Lockwood, 1980; Holeton *et al.*, 1982; Swift, 1983; Anders *et al.*, 2020) and other small pelagic fish (Marçalo *et al.*, 2006, 2010; Olsen *et al.*, 2012a; Tenningen *et al.*, 2012; Marçalo *et al.*, 2018), as well as current understanding of the general teleost stress response (Wendelaar Bonga, 1997).

Crowding mackerel invokes an avoidance response characterized by increased swimming activity (Anders *et al.*, 2019b). The lactate levels in this study indicate this behavioural response exceeded the anaerobic threshold. However, the time taken for mortalities to manifest, the physiological profile of moribund fish and the condition of injured/non-injured individuals suggests that exercise induced exhaustion and acidosis (either metabolic or respiratory) was not the primary cause of mortality in this study. Such occurrences would be expected within 4–8 hours after crowding and be marked by notable blood acidification (Wood *et al.*, 1983). Instead, the results suggest that fish died due to injury effects. It is probable that the increase in behavioural activity (Anders *et al.*, 2019b) in response to the small volume of crowded cages increased the likelihood of abrasive contact with netting and other fish. Mackerel as a species may be particularly susceptible to skin injuries due to their thin integuments (Kitsios, 2016). Skin injuries in fish have been shown to provoke endocrine stress responses and compromise osmoregulation (Gadomski *et al.*, 1994; Olsen *et al.*, 2012a; Cook *et al.*, 2019a), as was seen for moribund mackerel in the present study. Skin injury increases susceptibility to pathogen infection (Sveen *et al.*, 2020), which may be further exacerbated by cortisol’s inhibitory effect on immune responses (Pottinger, 2007). Under such circumstances, an inflammatory response to infection may result in a physiological profile similar to that observed for moribund fish in the current study (Bateman *et al.*, 2017; Miller *et al.*, 1980). Further evidence is seen in the elevated levels of K^+^, which can be indicative of cell damage (Gräns *et al.*, 2016). This, together with the observed reduction in moribund HCT values, suggests haemolysis occurred for some fish. We did not quantify the pathogenic load in the cages or investigate the histology of injured mackerel skin; both may be considered for future work to further explain mortality mechanisms. Differences in injury type and severity (perhaps arising from fish or net contact) should also be investigated.

The similarity in condition factors between dead and moribund fish precludes the possibility of post-mortem change. Rather, the observed differences in condition between injured and non-injured individuals suggest that skin injury resulted in a reduction in condition. Such whole-body, tertiary level responses have been noted in other pelagic species following fishing gear interaction (Suuronen *et al.*, 1996; Marçalo *et al.*, 2010; Olsen *et al.*, 2012a; Tenningen *et al.*, 2012) and are symptomatic of stress induced mobilization of energy reserves (Sopinka *et al.*, 2016), or perhaps a failure in foraging. The use of Fulton’s K is widespread and therefore allows comparison of species-specific condition across studies. However, its assumption that weight scales as a cube of length has been criticized and means that comparison between groups of individuals of different lengths may not be valid (Wuenschel *et al*., 2019). Any such effect can be expected to be negligible in the present study due to the similarity in mackerel length between trials. Even so, future work should consider applying a range of indices to gain a more in depth understanding of stress-related changes to allometric condition (Wuenschel *et al*., 2019).

### The effect of different degrees of crowding

All modelling procedures indicated highly significant effects of the ‘trial’ variable on the various physiological, injury and survival outcomes. For the trials in which survival was monitored, the probability of injury and mortality was positively correlated with increasing crowding density. The degree of physiological disturbance and time taken to recover to baseline generally followed the same pattern. For some parameters, the rate at which disturbance developed during crowding also correlated with density, and longer exposure resulted in a higher disturbance. Taken together, these findings demonstrate that the magnitude of mackerels’ physiological response to crowding is dependent on stressor intensity (in terms of density) and duration. The available results also allow the conclusion that the duration of any crowding-induced physiological disturbance, as well as the probability of skin injuries and survival, depend upon stressor intensity (i.e. crowding density). These stressor-response relationships are in accordance with previous studies on both mackerel and other small pelagics (Pawson and Lockwood, 1980; Holeton *et al.*, 1982; Lockwood *et al.*, 1983; Swift, 1983; Marçalo *et al.*, 2006; Huse and Vold, 2010; Marçalo *et al.*, 2010; Tenningen *et al.*, 2012). Importantly, the lack of mortality, coupled with the minimal physiological disturbance and low injury rates for the low crowding trial, indicate that some level of crowding is tolerable for mackerel. This agrees with the findings of Handegard *et al.* (2017).

### Confounding factors

There were a number of factors that may have confounded our results: (i) the higher pre-treatment condition of fish in the May/June trials, (ii) the warmer water temperatures in August/September and (iii) the presence of varying degrees of hypoxia during crowding between different trials. Mackerel naturally exhibit substantial seasonal variation in condition, with the poorest condition being found between March and May (Olafsdottir *et al.*, 2016). The high condition for May/June trials in this study therefore likely arose because of the fishes extended captivity time in the ‘holding cage’, which excluded many naturally occurring stressors and gave them longer access to highly nutritious feed pellets. Higher condition gives a greater capacity to cope with increased allostatic load (Moberg, 2000). This extra capacity may explain to some degree why no mortalities were recorded in the ‘Low’ trial (where condition factors were higher) despite the presence of injured fish. For bluegill sunfish (*Lepomis macrochirus*), lower condition fish had greater variability in corticosteroid responses to stress (Cook *et al.*, 2012), which may modify endocrine mediated physiological responses. Fish condition for the August/September trials was similar to that of non-captive individuals for the time of year (Olafsdottir *et al.*, 2016).

Temperature has a highly modifying effect of fish metabolic rates, physiological processes and ultimately survival (Gale *et al.*, 2013). Within species specific preferred ranges, increasing temperatures are typically associated with a reduction in survival. Simulated crowding of sardine (*Sardina pilchardus)* at 18°C or 23°C resulted in ~25% reduction in survival at the higher temperature (Marçalo *et al.*, 2010). Mackerel temperature preference is between 7 and 13°C (Nøttestad *et al.*, 2015). Fish in the August/September trials therefore experienced relatively high temperatures (16.1–17.9°C), not only compared to the May/June trials (10.9–13.9°C) but also to their preferred range. Although conducted at approximately the same time of year as the main mackerel fishery in Norway, temperatures for the August/September trials likely exceed what mackerel would experience during crowding events in the offshore purse seine fishery (11–14°C; our unpublished data). From the available data, it is not possible to determine if and how these confounding factors may have influenced the results of the current study, but it is reasonable to hypothesize that the increased temperatures in the August/September trials increased allostatic load and may have therefore reduced the survival potential of stressed and injured individuals. Even so, high survival was likely in the low density trial (92 kg/m^3^) in May/June, because it was similar to previously established safe thresholds for mackerel (88 kg/m^3^) (Handegard *et al.*, 2017).

The reductions in oxygen during crowding treatments probably arose due to limited water exchange in the densely crowded biomass of the school. A similar phenomenon is often observed in the fishery, where hypoxia can be more severe (~3 mg/l) when catch sizes are large (Breen *et al.*, 2020b). The presence of hypoxia in our trials therefore increased the realism of our simulations and, while unlikely to have been lethal, probably added to the crowding induced allostatic load. Hypoxia may therefore account for some of the between trial variation in responses we observed. As hypoxia was more severe in the moderate and high crowding trials, the threshold for anaerobic metabolism would be lower, inflating the development rate and magnitude of dependent parameters such as lactate and osmolality.

### Study limitations

Due to the inherent challenges of our sea-based mesocosm setup and the need for adequate control treatments, the various crowding densities were replicated only once (although there were two ‘high and prolonged’ trials, one suffered from missing data regarding the applied density). In some cases, we re-used the same group of fish in different trials. Potential variability in response between different groups of mackerel exposed to the same densities cannot therefore be examined using the available data, leading to uncertainty regarding the generalizability of the results. However, correlation between increasing density and the magnitude of the observed responses, coupled with the similarity in findings with previous studies (Pawson and Lockwood, 1980; Holeton *et al.*, 1982; Swift, 1983), would suggest our findings are still of value. With the set up and transfer methods described in the current study, it would be difficult to replicate exact densities across different cages because total biomass is not accurately known until the termination of the trial. However, graduated markings on the lower pyramidal section of the cages may assist with estimating available volume during future trials.

Although it would be unlikely to alter the conclusions of this study, the use of net cages (as opposed to a more controllable tank setup) resulted in a greater uncertainty as to when post-crowding mortalities occurred due to the relative difficulty of collecting dead fish. This effect likely partially explains the high variability in moribund fish physiological profiles (as some fish were closer to death than others, depending on when they could be removed from the cage). The use of feed pellets to encourage a feeding response and hook capture was unavoidable given our experimental arena. However, this methodology, along with natural variability in available prey items in the cages, may have led to differences in feeding status between fish or trials that could have influenced physiological responses (Bry, 1982). However, any effects would be likely marginal compared to the stressor induced effect (Olsen *et al.*, 2005, 2012b). The collection of fish by hook and handline may have selected for less satiated individuals or certain behavioural types (Klefoth *et al.*, 2017); factors that have been shown to determine physiological stress responses (Bry, 1982; Louison *et al.*, 2017; Koeck *et al.*, 2018). This may explain why we did not observe gross physiological disturbance in individuals apart from in moribund fish; feeding motivation can be expected to be reduced in non-coping individuals (Portz *et al.*, 2006) and thereby their availability for capture and probability of being included in our sample. If true, our results may underestimate the magnitude and frequency of disturbance for individuals following crowding. Samples collected during crowding periods should not suffer from this effect as fish were collected at random using a dip net. It is also possible that captivity habituation may have somewhat muted physiological responses (Schreck, 2000).

### Considerations for future work

Although our finding of skin injury being a cause of delayed mortality agrees with previous work (Pawson and Lockwood, 1980; Holeton *et al.*, 1982; Lockwood *et al.*, 1983; Swift, 1983), considerable differences in scale (and possibly stressor severity) exist between the simulations presented in this study and real purse seine capture scenarios (where individual catches can exceed 500 tonnes). For instance, varying catch sizes, net sizes, sea state conditions and gear operations between vessels and casts means that a wide range of crowding densities likely occur during real fishing. Due to the lack of empirical estimates of density from the field (Tenningen *et al.*, 2015), it is somewhat uncertain how well the densities applied in the present study reflect a real capture scenario. Our results are therefore best considered as a description of the directionality of response of mackerel to various degrees of crowding stress, rather than being an accurate representation of the magnitude of physiological response or mortality rates for the fishery.

In the only large-scale, at-sea mackerel slipping trials available in the literature (Huse and Vold, 2010), most deaths occurred within 2 days of the crowding treatment. This is markedly different from our findings and suggests mortality mechanisms are different in large fishery catches. The relatively small scale of our experiments may have meant that fish to net contact was a relatively probable event compared to a real capture scenario. Based on oxygen data from the fishery (Breen *et al.*, 2020b), it is reasonable to hypothesize that hypoxia may play a more important role in post-slipping mortality in large at-sea catches, where it can be expected to develop faster and be more severe than what was observed in the current study. Mackerel may be especially vulnerable to hypoxia as they are oxyphilic (Johnstone *et al.*, 1993). Consequently, hypoxia and its effects on physiology and mortality should be the focus of further work. However, the set up used in the current study does not allow crowding to be separated from the confounding effects of hypoxia (or temperature and fish condition). Further research is therefore needed on two scales: Firstly, tightly controlled laboratory tank experiments in which hypoxia can be applied independent of crowding and any modifying effects of temperature and condition examined. Secondly, large-scale slipping sea trials should be attempted (as described by Huse and Vold (2010)) to allow examination of stress responses and survival outcomes under more fishery-realistic conditions.

The extended time over which mortalities occurred in the present study may indicate that monitoring periods in previous studies examining mackerel post-crowding survival (Pawson and Lockwood, 1980; Holeton *et al.*, 1982; Lockwood *et al.*, 1983; Swift, 1983; Huse and Vold, 2010) may have been inadequate to capture all treatment related mortalities (the longest period was ~6 days; Swift, 1983). Mortality rates in these previous studies may therefore have been underestimated. We cannot, however, exclude this possibility in the present study either, due to the substantial numbers of injured but surviving fish and the demonstrated reduction in survival probability due to injury. Future work may therefore need even longer periods of survival monitoring.

### Conclusions and implications for welfare conscious fishing practices

Welfare conscious fishing practices are those that minimize physical damage and allostatic load on captured animals (Breen *et al.*, 2020a). Collectively, the results of this study indicate that crowding in a purse seine would likely cause increases to allostatic load, both in terms of physiological disturbance and skin injury. Following the release of unwanted catch (slipping), these effects may result in a loss of condition and mortality for a proportion of fish. However, the results also suggest that the magnitude, recovery profile and likelihood of these responses is dependent on crowding (and possibly hypoxia) intensity and duration. Furthermore, mackerel can tolerable low levels of crowding (<92 kg/m^3^).

The welfare of mackerel caught and released by purse seine in the fishery may therefore be best maintained by (i) reducing crowding density and its duration, (ii) minimizing abrasive contact and (iii) targeting high condition fish that would be best able to cope with increased allostatic load. These recommendations are supported by the results of this study in that (i) crowding density and/or its duration determined the magnitude of response, as well as the probability of injury and survival outcomes, (ii) skin injury (likely arising from abrasive contact) was demonstrated as a possible cause of death following crowding and (iii) injured and moribund/dead individuals had reduced condition factors relative to non-injured individuals.

Most Norwegian purse seine mackerel fishing occurs in September and October, specifically to target high condition (and therefore profitable) fish (Duinker and Pedersen, 2014; Sogn-Grundvåg *et al.*, 2019). The recommendation to target high condition fish is therefore already fulfilled to some extent. Abrasion and resulting skin injuries may be mitigated by alteration to net mesh configuration and material (Barthel *et al.*, 2003; Digre *et al.*, 2010) in the bunt end of the purse seine where crowding typically occurs. To further reduce the negative effects of crowding, fishers could target smaller schools to (i) reduce the likelihood of excess catch and therefore the need to slip and (ii) maintain low densities in the net. Good catch welfare practices during slipping should also ensure crowding levels do not exceed the bare minimum required to encourage fish to exit the net (Anders *et al.*, 2019a).

## Funding

This work was supported by the Norwegian Seafood Research Fund (Fiskeri- og Havbruksnæringens Forskningsfond, Project No. 901350).
